# Federated machine learning for a facilitated implementation of Artificial Intelligence in healthcare – a proof of concept study for the prediction of coronary artery calcification scores

**DOI:** 10.1515/jib-2022-0032

**Published:** 2022-09-05

**Authors:** Justus Wolff, Julian Matschinske, Dietrich Baumgart, Anne Pytlik, Andreas Keck, Arunakiry Natarajan, Claudio E. von Schacky, Josch K. Pauling, Jan Baumbach

**Affiliations:** Chair of Experimental Bioinformatics, TUM School of Life Sciences Weihenstephan, Technical University of Munich, Maximus-von-Imhof-Forum 3, 85354 Freising, Germany; Syte – Strategy Institute for Digital Health, Hohe Bleichen 8, 20354 Hamburg, Germany; Chair of Computational Systems Biology, University of Hamburg, Notkestreet 9-11, 22607 Hamburg, Germany; Preventicum Essen, Theodor-Althoff-Str. 47 45133 Essen, Germany; Preventicum Duesseldorf, Koenigsallee 11, 40212 Duesseldorf, Germany; Independent Researcher, Digital Health, Informatics and Data Science, Lower Saxony, Germany; Department of Diagnostic and Interventional Radiology, Klinikum rechts der Isar, Technical University of Munich, Ismaningerstr. 22, 81675 Munich, Germany; LipiTUM, Chair of Experimental Bioinformatics, TUM School of Life Sciences, Technical University of Munich, Maximus-von-Imhof-Forum 3, 85354 Freising, Germany; Computational BioMedicine Lab, Institute of Mathematics and Computer Science, University of Southern Denmark, Campusvej 55, 5230 Odense, Denmark

**Keywords:** artificial intelligence, coronary artery calcification, federated machine learning, privacy-preserving data processing

## Abstract

The implementation of Artificial Intelligence (AI) still faces significant hurdles and one key factor is the access to data. One approach that could support that is federated machine learning (FL) since it allows for privacy preserving data access. For this proof of concept, a prediction model for coronary artery calcification scores (CACS) has been applied. The FL was trained based on the data in the different institutions, while the centralized machine learning model was trained on one allocation of data. Both algorithms predict patients with risk scores ≥5 based on age, biological sex, waist circumference, dyslipidemia and HbA1c. The centralized model yields a sensitivity of c. 66% and a specificity of c. 70%. The FL slightly outperforms that with a sensitivity of 67% while slightly underperforming it with a specificity of 69%. It could be demonstrated that CACS prediction is feasible via both, a centralized and an FL approach, and that both show very comparable accuracy. In order to increase accuracy, additional and a higher volume of patient data is required and for that FL is utterly necessary. The developed “CACulator” serves as proof of concept, is available as research tool and shall support future research to facilitate AI implementation.

## 1 Introduction

### 1.1 Benefits and objectives

The real world usage of Artificial intelligence in healthcare routines is still in its beginning and several success factors for a facilitated implementation have been defined in the past [1]. One key success factor is the technological implementation, as the data access and algorithm training is crucial in order to generate reliable results. In our study a federated and centralized machine learning model approach for the early detection of coronary artery diseases (CADs) were compared with each other.

Physicians commonly assess the CAD risk of patients based on coronary artery calcification scores (CACSs), obtained via non-contrast computed tomography (CT) scans. However, CT produces high costs and exposes patients to radiation and, hence, unnecessary scans must be avoided. Therefore, an effective decision support tool predicting coronary artery calcification (CAC) risks and, thus, CT necessity can provide significant benefits.

In this study we aim to analyse (1) whether it is possible to predict CACSs with Artificial Intelligence (AI) and (2) how a privacy-preserving machine learning (ML) approach based on federated data sources, namely federated machine learning (FL), performs as compared to a conventional ML approach that is based on centralized data. Based on these results, improvement options for the particular indication and FL in general are discussed.

### 1.2 Coronary artery calcification scores (CACSs)

Cardiovascular diseases are the leading cause of death worldwide and coronary artery diseases (CADs) represent the leading cause of cardiovascular mortality [2, 3]. In order to counteract its significant mortality rates, treatment regimens should be adapted as early as possible [4]. For individuals with no symptoms or known pre-existing conditions, various factors (e.g. age, behavioral characteristics) can be used to determine the risk of a cardiovascular disease and increased CAC levels have been found to be significantly positively associated with cardiovascular diseases [4]. In this regard, prior research revealed that 27% of radiologists in the US already rely on CAC CT scans on a regular basis, making it the most common type of CT scan in the country [5]. This does not seem surprising in light of the fact that there are guidelines recommending CAC CT screening for asymptomatic men aged 45–75 and woman aged 55–75 (except for those with very low risk) [5–7]. This translates into c. 30 million citizens being generally eligible for CAC CT screening in the US [8, 9]. However, many of them may not actually have elevated CACSs, which causes the discussion on whether too many CACS CT scans are conducted [10].

### 1.3 Application of Artificial Intelligence for CACSs prediction

Considering this risk/benefit trade-off, we assessed whether an AI-based decision support tool could potentially contribute to reducing cases of CAC CT screening where patients do not actually have elevated CACSs. To this end, we used datasets from two medical institutions with a total of 1450 patients and analyzed the following four independent CAC risk factor areas – those are also in detail explained in the supporting information section:

(I) Age and biological sex
(II) Obesity (measured through waist circumference)
(III) Dyslipidemia (measured through cholesterol, triglycerides, high-density lipoprotein HDL, low-density lipoprotein LDL)
(IV) Diabetes mellitus (measured through HbA1c)

Further potential risk factors were not included, as the selected factors are (A) reflecting some of the major named risk factors in literature and (B) the algorithm should serve as a tool for physicians in daily routine [11].

We used AI for the purpose of our study as AI can lead to significant benefits in healthcare and has, for example, already proven to be valuable in the area of cardiology, but also in the context of other medical indications, such as Covid-19 as well as of personalized treatment [12–14].

However, conventional AI approaches generally require access to large amounts of data to achieve precise prediction and are, therefore, often not easy to be implemented. To circumvent this issue, the concept of FL can be applied as this enables data access across different units without requiring the exchange of raw data, thus, maintaining data privacy. Therefore, besides assessing whether AI can generally be useful to predict CACSs, we assessed how FL as a privacy-preserving ML approach performs as compared to a conventional ML approach.

The concept of FL goes back many decades, where it did not initially relate to AI, but was supposed to assist in the discovery and access of learning content from the diverse collection of content repositories [15]. Only in 2017, Google proposed to apply the concept of FL in the ML context where the main idea was to generate ML models based on datasets that are distributed across multiple devices, while at the same time preventing data leakage [16].

Today, the term FL refers to AI model training based on multiple local datasets, yet without the exchange of the participating units’ primary data but instead only model parameters. The key goal is to enable multiple actors to jointly build an aggregated ML model without facing the difficulties of data sharing [17].

Altogether, an FL model represents a decentralized ML approach where the participating units’ respective ML models are locally trained in a first step, and the model parameters are subsequently shared by all units, commonly via a central coordinating server, and merged into an aggregated model [18, 19]. Consequently, FL can provide significant advantages as compared to conventional ML approaches as the privacy-sensitive medical patient data must not be shared across participating units, enabling FL applications even where data-related restrictions prevent the application of conventional ML approaches.

There are already some first FL applications in healthcare, for example, in the context of the prediction of mortality and hospital stays, the medical diagnosis classification of diabetes and heart failure, and the prediction of the hospital readmission risk [20–23]. One major real world example is Owkin, a startup which has raised >300 Million USD in funding for their federated learning platform and which works for example in their EU project Melloddy in a collaboration with 10 pharmaceutical companies on a federated learning driven drug discovery process with molecule data [24]. For the described CACS prediction, an AI model based on risk factors for elevated CACSs could serve as a valuable decision support tool and in order to circumvent data privacy challenges, the benefits of FL shall be leveraged.

## 2 Materials and methods

To apply FL, we used the FeatureCloud platform, which targets to simplify privacy-preserving access to comprehensive medical data across medical institutions [25]. FeatureCloud, which is publicly available under http://featurecloud.ai, is supported by the European Union Horizon 2020 program and was developed as a joint effort between several European universities and companies, including the Technical University of Munich, the Philipps University of Marburg, the University of Maastricht, the Medical University of Graz, the University of Hamburg, the University of Southern Denmark and Gnome Design SRL, Romania.

**Figure 1:**
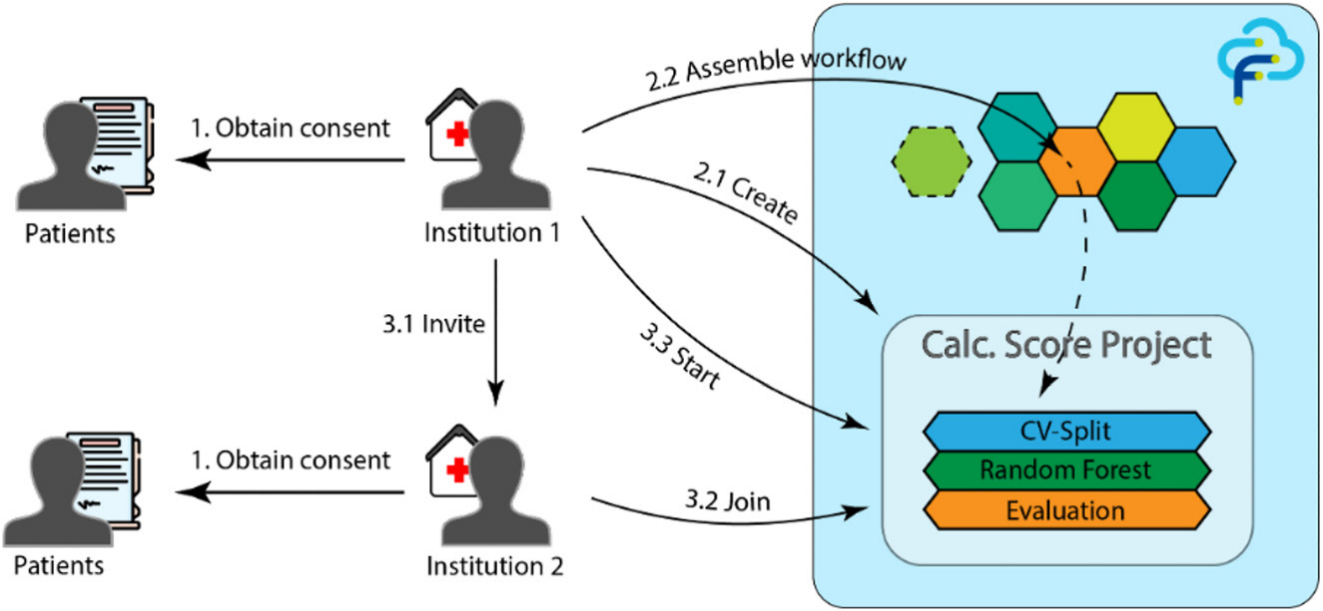
Two institutions used the FeatureCloud platform and institution 1 has been the FL project leader (Authors).

After the FeatureCloud app has been installed by all participating medical institutions, the procedure is as follows – as also illustrated in Figure 1:

1. Obtaining patients’ consent for the usage of their anonymized data
2. Creation of the FL project in FeatureCloud and assembling the workflow
3. Inviting other medical institutions to participate and starting the project

Since we aimed at shedding light on whether it is possible to predict CAC with conventional AI and FL as a privacy-preserving ML approach performs as compared to a conventional ML approach, an according FeatureCloud app has been developed and can be accessed via https://featurecloud.ai/ai-store/71. FeatureCloud is an integrative solution since multiple medical institutions can join the platform to share their data for AI model training and thus by each new institution the ML training data set is increased and higher prediction accuracies or additional prediction usecases can be defined – currently there are already more than 20 applications available on the FeatureCloud application store.

### 2.1 Study design – conventional AI for CACS prediction

As to the first research question, we trained the algorithm for the decision support tool using an anonymized dataset of the above mentioned two medical institutions that in total comprises 1450 patients. The pre-processing resulted in 680 remaining patient samples. For the centralized ML approach, the AI model was trained based on the entire dataset, i.e. on all patient samples of the two medical institutions, as if the data stemmed from the same source in the first place. Further details can be found in the supporting information section.

### 2.2 Study design – FL for CACS prediction

In addition, we analysed how FL performs as compared to a conventional ML approach, namely whether a similar performance in terms of sensitivity and specificity can be achieved when the AI model is trained in an FL (rather than a centralized) setting.

The local data is pre-processed by each medical institution before the local model training takes place. The model parameters are subsequently shared by both medical institutions and merged into an aggregated ML model. This aggregated model is then shared with both institutions for their own use. During the entire process, FeatureCloud does not require any transfer of the medical institutions’ underlying primary medical data, but only the exchange of model parameters. Accordingly, in our particular use case, participating physicians would not need very extensive data of their own, but could use the aggregated ML model to predict patients’ CACSs and, based on this, assess the need for CAC CT screening.

**Figure 2:**
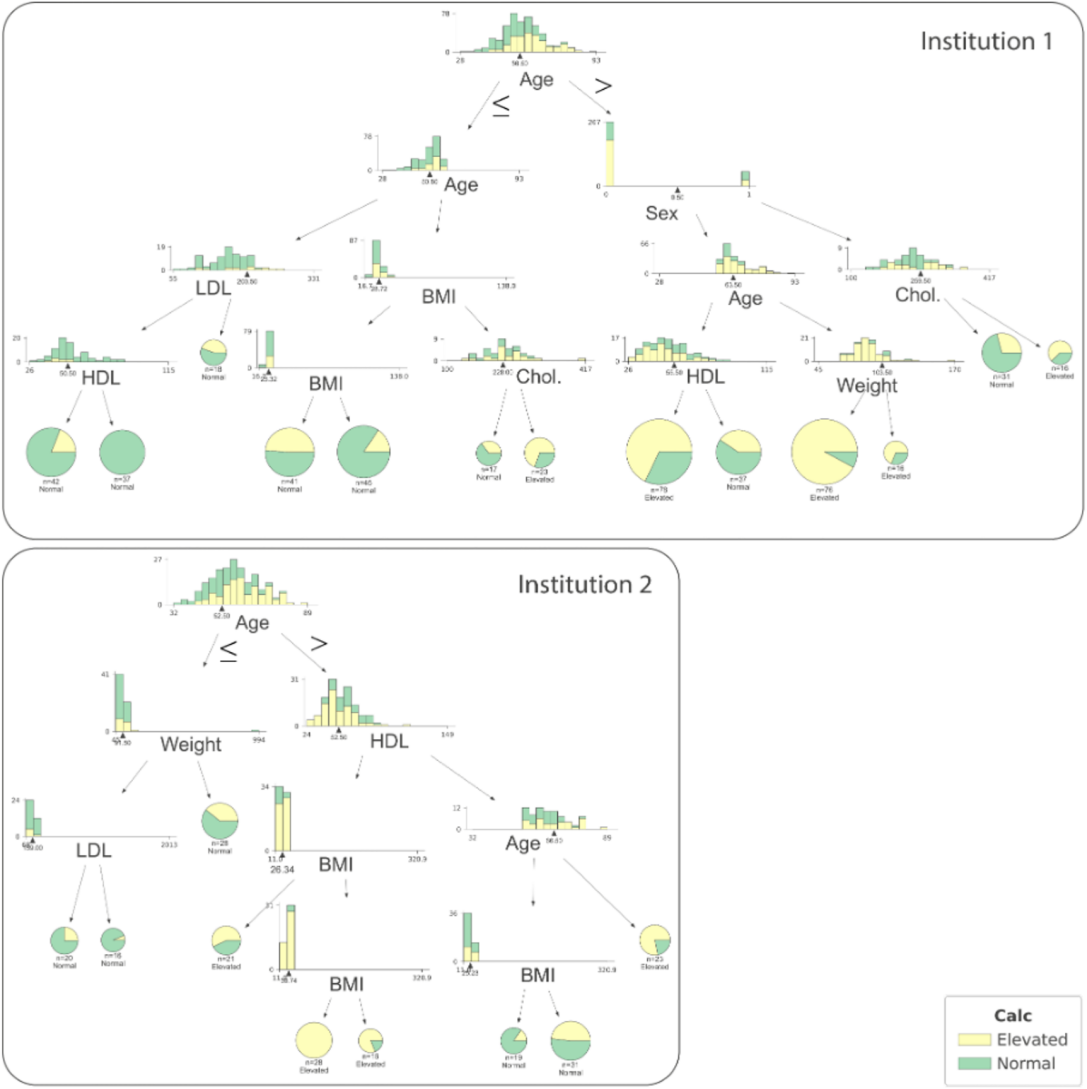
Decision trees trained with the data from institution 1 and 2; both trees are first split by age, but already diverge at the next level (Authors).

In order to demonstrate the likelihood analysis of a CACS ≥5, we trained two random forest models locally, i.e. based on each of the medical institution’s own local dataset. The resulting decision trees are visualized in Figure 2.

The parameters of the locally trained AI models from both medical institutions are aggregated into one joint FL model, via sharing of the local model parameters rather than the medical institutions’ primary data as it would be required when applying the conventional ML approach. Additional information regarding the training process is described in the following section.

For the conventional centralized ML model, a random forest model with all 680 patient samples (i.e. from both medical institutions) was trained. For the FL model, an aggregated random forest model was computed based on the model parameters of the locally trained models of the two medical institutions with 477 and 202 samples, respectively.

We pre-processed our data in terms of removing samples with missing values and transforming the continuous CACSs into two categories: Elevated and normal, representing values above or equal (CACS =>5) and below a CACS threshold of 5 (CACS <5), respectively. We investigated how the results change when replacing missing values with their mean or median values to allow for using incomplete samples during the training of the random forest model without distorting the results too heavily. This led to no improvement and was, therefore, not pursued for the subsequent steps. After removing the incomplete samples, 680 patients remained. The score threshold of 5 was chosen because it both indicates a health risk and divides the dataset into two equally large groups of 340 patients each.

For the classification, we selected a random forest model for our analysis as such models are frequently used as classifiers due to their ability to cope with categorical and continuous features alike and due to their general versatility. To conduct an FL analysis with the two participating medical institutions, we first trained a random forest at each institution locally using only their local data. To obtain the aggregated model, the decision trees of both random forests were merged into one random forest model. Institution 1 and 2 contributed 67 and 33 decision trees, respectively, proportional to their number of samples. During training, gini index minimization was used for splitting and the number of selected random features for each tree was 4.

**Figure 3:**
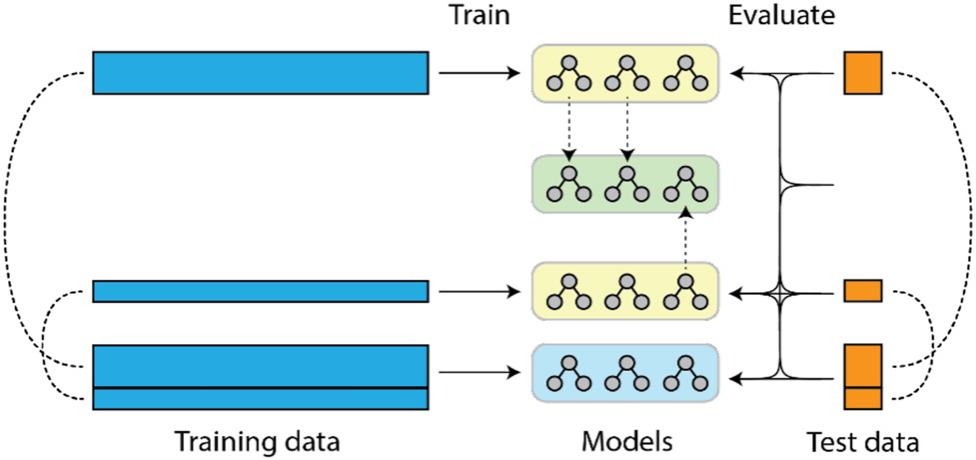
Training and evaluation of the FL model.

To evaluate the models, we developed a tailored cross-validation which takes all possible combinations of splits into consideration (see Figure 3). This type of cross-validation was performed ten times to further reduce random effects in the evaluation, leading to 1000 test runs in total.

The yellow random forests have been trained on the local data only. The green random forest model emerges from aggregation of the local random forest models. To reflect the different size of the training data, Institution 1 contributes twice as many trees to the aggregated model as Institution 2. The blue reference model is the conventional centralized ML model, which was trained on the whole dataset, i.e. of both institutions.

**Figure 4:**
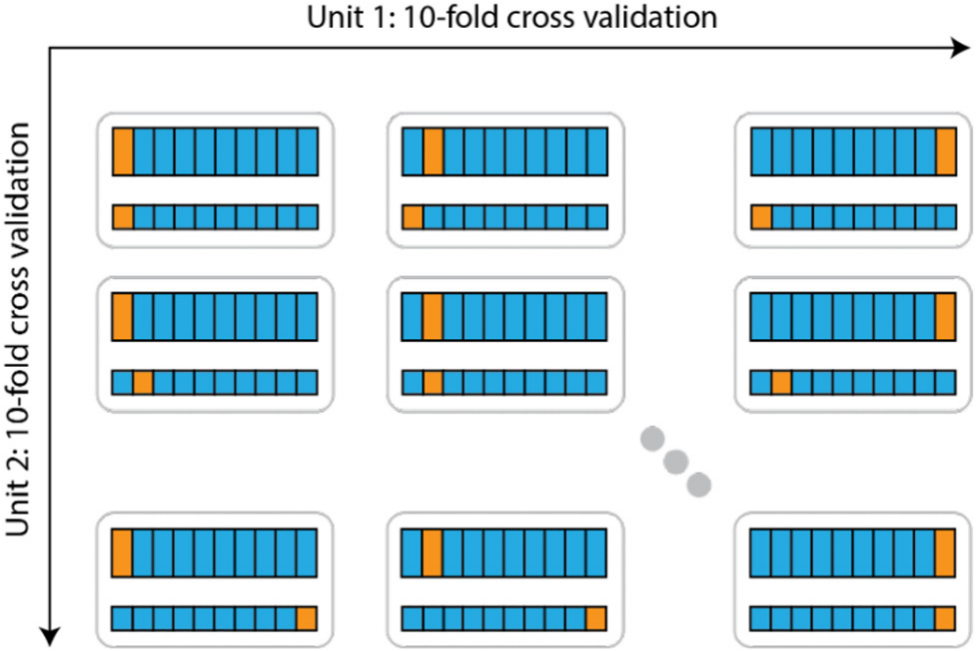
Cross-validation in the FL setting.

Each institution performed ten even splits on their data, as illustrated in Figure 4. To assess the performance of the aggregated model, each combination of splits from both institutions was used for validation, mirroring the conventional cross-validation approach in an FL federated setting, leading to 100 cross-validation steps.

The application is available on the FeatureCloud AI Store and allows for running the analysis for an arbitrary number of participants. Each participant contributes a number of decision trees proportional to their share of the total data. A web frontend allows for checking the number of valid samples and monitoring progress. The final accuracy, obtained through cross-validation, is displayed as well. The global random forest, consisting of all decision trees, can be downloaded by each participant for further evaluation or to predict CAC classes on unseen data.

## 3 Results

**Figure 5:**
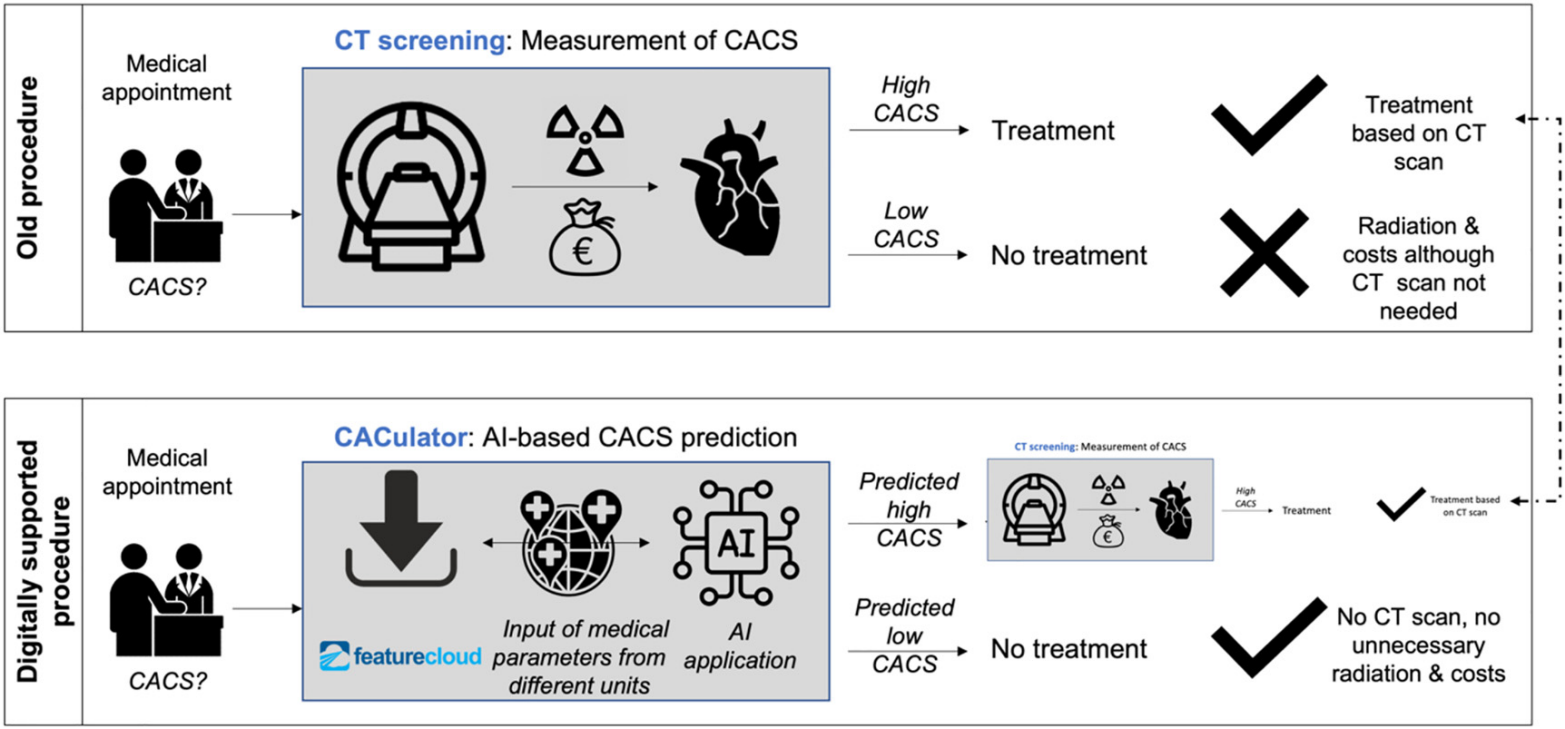
Comparison of the “analogue” and “digitally” supported CACS patient assessment and according benefits (Authors).

It has been assessed if and to what extend the existing “classical” analogue CACS assessments via CT screening could be improved through an AI based prediction model and the according processes as well as potential benefits are displayed in Figure 5:

The results of both, the centralized ML and the FL approach and the respective sensitivity and specificity values are displayed in Table 1 and the median values of all patient characteristics are tabulated in Table 2:

**Table 1:** Results of the centralized and FL analysis, showing the mean accuracy, ROC AUC, sensitivity, specificity and number of patient samples for Institution 1 and 2 individually, the FL model and the centralized ML model.

Metric	Institution 1	Institution 2	Centralized	Federated
Accuracy	68.12%	64.71%	67.65%	67.65%
ROC AUC	75.22%	71.14%	75.52%	75.09%
Sensitivity	64.86%	66.67%	65.52%	66.67%
Specificity	71.61%	63.64%	69.70%	68.57%
Samples	477	202	680	477 + 202

**Table 2:** Median values for age, height, weight, waist, cholesterol, triglycerides, HDL, LDL, HBA and BMI in the complete group, the group of patients with a CACS below and above 5, and the patients from Institution 1 and Institution 2 individually.

	All	CACS < 5	CACS ≥ 5	Inst. 1	Inst. 2
Age	57.0	54.0	61.0	57.0	57.0
Height	179.0	179.0	178.0	179.0	178.0
Weight	87.0	86.0	87.0	87.0	86.0
Waist	98.0	96.0	101.0	99.0	98.0
Chol.	228.0	230.0	224.0	228.0	224.5
Tri.	129.0	122.0	136.0	127.0	131.0
HDL	51.0	53.0	49.0	50.0	53.0
LDL	156.0	158.0	152.0	160.0	149.5
HBA	5.5	5.4	5.6	5.5	5.5

As visible, it can be derived that the centralized ML and the FL random forest models showed similar sensitivity of 65.5% and 66.7% as well as specificity of 69.7% and 68.6%, respectively.

With regard to the main research questions, the following conclusions can be drawn:

(1) It is technically possible that an AI model is used for the prediction of CACS, which itself is based on the five risk factors age, sex, obesity, dyslipidemia and diabetes. Although the sensitivity and specificity is with ca. 70% not very high and further factors for improvement need to be identified and included, such an algorithm can already provide some value add, since existing CAC CT scan assessments entail a significant risk of erroneous patient risk group classification.
(2) The FL-based decision support tool would essentially be comparably useful as both of the approaches, the centralized and FL approach, deliver very similar outcomes in terms of sensitivity and specificity. However, given FL’s significant advantage of allowing privacy-preserving data access without actual primary data sharing across different institutions, FL might often represent the much more feasible approach in daily practice. Thus, FL can provide a significant benefit for future AI model training, especially where data is privacy-sensitive and scattered across medical institutions.

## 4 Discussion and conclusion

This publication focused on the data access and algorithm training through federated machine learning using the example of CACS prediction. While our results suggested that an AI-based decision support tool can generally be constructed and that the performance of the FL approach is similar to the one of the conventional ML approach, it should be highlighted that we did neither aim at creating a completely new risk indicator for the measurement of CACSs, i.e. choosing the optimal combination of risk factors, nor at replacing CAC CT scans or other assessment processes currently undertaken by physicians. Especially for the second one, a significantly higher accuracy of a prediction model would be required.

The obtained results with sensitivity and specificity levels of close to 70% still require further improvement in order to actually enable true benefits in clinical practice; suggestions to achieve such improvements are presented as part of this discussion. Thus, in the following, (1) an embedding of the work in the research landscape, (2) improvement areas for the developed FL CACS prediction model, and (3) overarching facilitators for FL application are discussed and the results are aggregated in a conclusion and outlook.

### 4.1 Research landscape

Prior studies also reflected on FL and for example Chamikara et al. highlighted that FL can require additional measures to guarantee data privacy or that computational IT bottlenecks can occur and thus supporting algorithms inside the FL platform can be applied [26].

One of these privacy measures can be so called “Differential Privacy”. Adnan et al. have analyzed The Cancer Genome Atlas dataset, which is particularly interesting since the images are derived through diverse imaging methods as well as devices and are marked differently. Since, as mentioned, FL can not provide a privacy guarantee because private information could in theory also be traced based on the shared model parameters, they applied differentially private FL. This framework quantifies the privacy of provided protocols and thus focusses on extracting as much information as possible while consuming the least privacy [27].

Also the accuracy difference between centrally trained models and FL has been analyzed and Kirienko et al. described in their literature research covering 26 publications that the prediction accuracy between both approaches is “equal” [28].

This has also been confirmed when FL was specifically applied for data access of electronic health records. Dang et al. could show that it was possible to apply FL for in-hospital mortality prediction by analysing gender, ethnicity, lab tests for blood urea nitrogen, the Glasgow Coma Scale, surgery type, heart rate, blood pressure and others out of in total 82 parameters for their model training [29].

In addition to that, Zerka et al. mentioned in their research that the convincing of medical centers to join an FL approach can be a hurdle and that also regulators need to be actively involved as suitable safeguards, since also with FL it remains a significant task to collect enough patient samples for ML training [30].

Furthermore, the analysis of CAC systems with support of technology has been researched in the past. Rogers and Aikawa for example focussed on both, the advances in molecular imaging and big data technology to map the disease more comprehensively. They described that AI models can improve diagnostics and risk assessment and that they could include additional biological data like vesicle release, mineral deposition or inflammation data as well as the analysis of omics data, which can contribute to a CAC development [31].

In addition to that, Sandstedt et al. evaluated an AI based CACS software for the scanning of tomography scans and came to the conclusion that calcified lesions could be accurately detected by the AI compared with conventional methods. They also highlighted that AI could be a crucial success factor for this global disease burden and that it should be applied in the real world clinical setting [32].

Our publication focussed not on a tool for the assessment of diagnostic scans, but medical parameters as input factors that could even be analyzed before a CT scan is conducted and thus potentially the algorithm can enable patient screening without exposure to radiation and its according costs. The prediction accuracy of 70% is not yet high enough for a clinical day to day implementation and improvement areas for an increased prediction capability are described in the following paragraph.

### 4.2 Improvement areas for FL-based CACS prediction

The accurate FL prediction of elevated CACSs could provide a very meaningful value-add, as this would allow to adopt treatment regimens at an early stage.

There are various means by which the model’s prediction power could be further improved, both quantitatively and qualitatively:

(1) Quantitative enhancement measure: An extension of the 1,450 patient collective, out of which only 680 patient samples could be included after data pre-processing, could improve accuracy. This could be achieved through the inclusion of additional medical institutions which grant access to their respective patient data.
(2) Qualitative enhancement measure: Several medical parameters can influence CACS and the prediction accuracy of the trained AI algorithms demonstrates, that further parameters are required e.g. from medical practice and laboratories. The prediction model’s accuracy could be improved through the inclusion of these additional CAC risk factor areas and extension examples could be family history, diet, hypertension, smoking, chronic kidney disease, psychosocial factors, elevated lipoprotein and elevated apolipoprotein [33, 34].

Both of these measures shall be addressed through open access to the CACulator app on the FeatureCloud website (https://featurecloud.ai/ai-store/71). The input of additional and new model parameters by various medical institutions shall be fostered through this international research collaboration.

Furthermore, the developed FL model could be compared with other scores for the identification of cardiovascular risk, but the American College of Cardiology and the American Heart Association published in their review, that current risk scores were found to vary widely with regard to the populations from which they were derived, risk marker inputs/covariates, and outcomes of interest [35]. One example is the ASCVD score – a calculation of the patient’s 10-year risk of having a cardiovascular problem, such as a heart attack or stroke, but also this scores has pitfalls, like overestimation of 10 year risk [36].

### 4.3 Overarching facilitators for FL application

In addition to that, there are more general improvement areas that could increase the real-world application of FL in healthcare, irrespective of this particular use case:

First, easy access to the software should be ensured by implementing it into daily medical routines, e.g. in the form of a smartphone application or by integration into the electronic health record (EHR) system of the participating medical institutions. To this end, several practical challenges need to be overcome. For example, the software would need to comply with the respective regulatory requirements, such as the GDPR in Europe or the FDA guidelines in the US, as well as medical product class guidelines. Furthermore, the solution would need to fulfil the requirements for app downloads (e.g. in the Google Playstore or Apple’s App Store) and the requirements of EHR providers (e.g. Cerner or Siemens).

An additional practical challenge for the application of FL is that its implementation requires human and financial resources on the clinical side and is, thus, also a business/economic decision. In order to also provide a compelling case from this perspective, the economic benefits and costs of implementing the FL solution should be assessed and measured. Such quantitative evidence would often contribute to a faster scaling of the respective solution and additionally pave the way for public grants and for better access to external resources [37]. This commercial aspect is also reflected in the fact that FL research is currently mostly driven by large and primarily tech-oriented industry players and not academia [38]. An increased amount of research by industry-independent scientific institutions would likely entail a number of benefits, including increased trust and application levels.

Furthermore, when applying FL in this research project, we also identified several administrative steps that would significantly contribute to achieving higher levels of FL application in real-world contexts in the future:

(1) Implementation: The first installation of FeatureCloud or a similar solution has to comply with legal standards, e.g. in the form of a contract between the medical institution and the software provider. This process should follow an automatized legal compliance process adapted to every country’s legal framework. It could even differentiate between research and operational implementation [30]. Furthermore, the software installation needs to be compatible with the local IT infrastructure specifications (e.g. firewalls) of the participating medical institutions. To avoid technical issues and lengthy and complex adaptation processes as well as mitigate the risk of delays, respective guidelines are required and should be made available upfront.
(2) Understandability: The processes for the application of the different ML models should be easy to understand and designed in a way for them to be initiated as well as steered by the participating medical institutions’ staff. To this end, explanations and training materials need to be provided by the FL provider. Potentially, even governmental institutions may get involved and support this process as this could further increase the trust level as to the application of AI in healthcare.
(3) Scaling: AI can and should be applied in the context of different medical indications. Patient data could be collected and, at best, be evaluated automatically for different medical indications (e.g. asthma, diabetes and electric implant monitoring). This requires the input of key decision-makers on medical and business management level and, as a basis for their decision-making, an objective and comprehensive overview of the opportunities and risks of AI in healthcare is needed. This could be enabled by a simple-to-use “entry point”, such as a functionality that evaluates existing datasets with regard to their applicability for an AI use case.

Progress in these improvement areas, namely the practical IT implementation in existing EHR structures, measurement of medical and economic impact as well as administrative support tools for implementation, understandability and scaling could significantly contribute to an increased usage of FL.

## 5 Conclusion and outlook

An overall facilitated implementation of AI in healthcare depends on several factors, but with regard to technological implementation, the access to data for the application of algorithms is crucial. This publication focused on the data access and algorithm training through federated machine learning using the example of CACS prediction.

It can be concluded that, AI can be used for CACSs prediction with a moderate accuracy using the mentioned five data points (age, sex, obesity, dyslipidemia, diabetes) and that a decentralized FL approach, which allows for privacy-preserving data access across medical institutions, demonstrated a similar performance as a conventional ML approach.

The current model performance of the proof of concept is still too limited for a clinical setting and further improvements are needed to allow for clinical implementation. The prediction accuracy of the CACS model could be improved through (a) a more comprehensive patient collective and (b) an extension to further CAC risk factor areas. These improvements require data access across institutions and are only possible in a privacy preserving context, thus federated machine learning can be a very important success factor regarding AI application for CACS prediction.

In addition to the use case-specific improvement areas, we also identified several general improvement areas that could increase the real-world application of FL in healthcare: First, medical institutions should be able to access the FL software in the context of daily medical routines and electronic patient records. Second, a reliable economic impact assessment is needed to support the strategic decision to apply FL, especially given the required medical team and financial resources. Third, administrative support in terms of legal and technological implementation standards, training material for the participating medical institutions’ staff and simple data validation mechanisms for the verification of AI suitability can facilitate implementation.

Overall, it can be concluded that FL can provide significant value across different indications as it allows to exploit data from different sources in AI model training via its privacy-preserving design and can thus support an overall increased implementation of AI in healthcare.

## Appendix

### A.1 Medical parameters

This section covers the relationship between the included models parameters and the CACS and outlines our rationale for including them in our prediction model:

#### A.1.1 Age and biological sex

The extent of CAC is strongly associated with age in men and women. Calcification is first detected in most men at around age 40, whereas women first show calcification around age 50 [39]. The incidence in women is delayed by 10 to 15 years as compared to men, likely attributable to the protective effects of estrogen [40].

The effect of estrogen and, also, according therapy on CAC has been examined, for example, for women aged 50 to 59 years during a 7-year period of treatment. The CAC was significantly lower for women assigned to estrogen substitution as compared to those receiving placebo [41].

#### A.1.2 Obesity (measured through waist circumference)

Further, obesity was shown to be strongly associated with an elevated risk of chronic heart diseases across several clinical studies [42–47] In this regard, the waist circumference, rather than the body mass index, is more likely directly associated with mortality [48–50].

#### A.1.3 Dyslipidemia (measured through cholesterol, triglycerides, HDL, LDL)

A prior study assessing CAD risk scores for 2,599 participants of the Dutch-Belgian Lung Cancer Screening Trial revealed nominally significant associations for genetic risk scores of low-density lipoprotein-cholesterol, total cholesterol, and obesity [48].

Furthermore, in a multi-ethic cross-sectional analysis, 4,917 atherosclerosis participants were classified into six groups defined by specific LDL-c, HDL-c, or triglyceride cut-off points. Multivessel CAC was defined as the involvement of at least two coronary arteries. This study revealed that all groups except for the one exhibiting hypertriglyceridemia had statistically significant prevalence ratios of having multivessel CAC as compared to the normo lipidemia group [51].

#### A.1.4 Diabetes mellitus (measured through Hba1c)

CAC tends to be higher in diabetic patients and represents an independent risk factor for adverse outcomes [52, 53]. In different studies, the average CACS for subjects with and for those without diabetes was 281 +/− 567 and 119 +/− 341, respectively. Also, the death rate was 3.5% and 2.0% for subjects with and without diabetes, respectively. In a risk-factor-adjusted model, there was a significant interaction of CACSs with diabetes, indicating that, for every increase in CACS, there was a greater increase in mortality for diabetic than for non-diabetic subjects. However, patients suffering from diabetes with no CAC demonstrated a survival rate similar to the one of individuals without diabetes and no detectable CAC (98.8% and 99.4%, respectively).
